# Multidrug-Resistance of *Vibrio* Species in Bivalve Mollusks from Southern Thailand: Isolation, Identification, Pathogenicity, and Their Sensitivity toward Chitooligosaccharide-Epigallocatechin-3-Gallate Conjugate

**DOI:** 10.3390/foods13152375

**Published:** 2024-07-27

**Authors:** Mruganxi Harshad Sharma, Suriya Palamae, Mingkwan Yingkajorn, Soottawat Benjakul, Avtar Singh, Jirayu Buatong

**Affiliations:** 1 International Center of Excellence in Seafood Science and Innovation, Faculty of Agro-Industry, Prince of Songkla University, Hat Yai, Songkhla 90110, Thailand; 6511030025@psu.ac.th (M.H.S.); suriya.pal@psu.ac.th (S.P.); soottwat.b@psu.ac.th (S.B.); jirayu.b@psu.ac.th (J.B.); 2 Department of Pathology, Faculty of Medicine, Prince of Songkla University, Hat Yai, Songkhla 90110, Thailand; mingkwan.y@psu.ac.th

**Keywords:** *Vibrio* spp., bivalve mollusks, *β*–hemolysis, biofilm formation, multidrug resistance, chitooligosaccharide

## Abstract

*Vibrio* spp. is a Gram-negative bacteria known for its ability to cause foodborne infection in association with eating raw or undercooked seafood. The majority of these foodborne illnesses are caused by mollusks, especially bivalves. Thus, the prevalence of *Vibrio* spp. in blood clams (*Tegillarca granosa*), baby clams (*Paphia undulata*), and Asian green mussels (*Perna viridis*) from South Thailand was determined. A total of 649 *Vibrio* spp. isolates were subjected to pathogenicity analysis on blood agar plates, among which 21 isolates from blood clams (15 isolates), baby clams (2 isolates), and green mussels (4 isolates) showed positive *β*–hemolysis. Based on the biofilm formation index (BFI) of *β*–hemolysis-positive *Vibrio* strains, nine isolates exhibited a strong biofilm formation capacity, with a BFI in the range of 1.37 to 10.13. Among the 21 isolates, 6 isolates (BL18, BL82, BL84, BL85, BL90, and BL92) were *tlh*-positive, while *trh* and *tdh* genes were not detected in all strains. Out of 21 strains, 5 strains showed multidrug resistance (MDR) against amoxicillin/clavulanic acid, ampicillin/sulbactam, cefotaxime, cefuroxime, meropenem, and trimethoprim/sulfamethoxazole. A phylogenetic analysis of MDR *Vibrio* was performed based on 16s rDNA sequences using the neighbor-joining method. The five MDR isolates were identified to be *Vibrio neocaledonicus* (one isolate)*, Vibrio fluvialis* (one isolate) and, *Vibrio cidicii* (three isolates). In addition, the antimicrobial activity of chitooligosaccharide–epigallocatechin gallate (COS-EGCG) conjugate against MDR *Vibrio* strains was determined. The minimum inhibitory concentration (MIC) and minimum bactericidal concentration (MBC) of COS-EGCG conjugate were in the range of 64–128 µg/mL. The antimicrobial activity of the conjugate was advocated by the cell lysis of MDR *Vibrio* strains, as elucidated by scanning electron microscopic images. *Vibrio* spp. isolated from blood clams, baby clams, and Asian green mussels were highly pathogenic, exhibiting the ability to produce biofilm and being resistant to antibiotics. However, the COS-EGCG conjugate could be used as a potential antimicrobial agent for controlling *Vibrio* in mollusks.

## 1. Introduction

Food contamination with pathogenic bacteria, viruses, parasites, or poison is the leading cause of foodborne diseases [1]. Foodborne illness continues to be a severe public health issue worldwide, including in Thailand. Outbreaks of illness caused by eating contaminated seafood have been frequently reported in Thailand. The consumption of contaminated seafood can lead to hospitalization and mortality, particularly in underdeveloped and poor countries [2]. Each year, 600 million cases of foodborne illness and 420,000 fatalities occur worldwide due to contaminated foods. Currently, 7.8 billion people live on the globe, and 56 million pass away each year and among them approximately 7.5% of global mortality results from foodborne illness [3]. Foodborne infection caused by one or more species of the Gram-negative *Vibrio* genus of bacteria, which has a curved-rod form, is typically linked to consuming undercooked seafood [4]. Environmental waterbodies that are brackish, and estuaries, frequently harbor pathogenic species from the genus *Vibrio*. Therefore, the public health implications of seafoods contaminated with these species are significant [5]. According to a survey carried out by the Centers for Disease Control and Prevention (CDC), it was found that every year in the United States, out of 80,000 cases, 52,000 cases are of vibriosis, which is caused by eating food contaminated by *Vibrio* species [6]. The CDC also mentioned that pathogenic *Vibrio* species may cause outbreaks and infections such as watery diarrhea, abdominal cramps, nausea, vomiting, fever, and chills. In some cases, this causes wound infections, bacteremia, and sepsis, which are the biological risks that seafood may harbor. Three species of *Vibrio* are responsible for foodborne diseases in seafood, and those species include *Vibrio parahaemolyticus, Vibrio vulnificus,* and *Vibrio cholera* [7]. Thus, the identification of foodborne bacteria in seafood, especially in mollusks, which can accumulate bacteria to a large extent due to their filter-feeding habits, is required.

In Thailand, mollusks, namely, blood clams (*Tegillarca granosa)*, baby clams (*Paphia undulata*), and Asian green mussels (*Perna viridis*), are consumed widely. Blood clams are widely distributed in the Indo-Pacific and are associated with the Arcidae family of the Mollusca phylum. Fresh raw blood clams are nutritious and contain 19.48% protein, 2.50% fat, 74.3% water, and 2.24% ash. These clams are known to be filter feeders, which makes them susceptible to microbial contamination and to causing foodborne diseases [8]. Baby clams belong to the Veneridae family found in the Indo-West Pacific on the nearshore shallow sandy substrate. With a crude protein content of around 68.77%, baby clams are a good source of protein [9]. Asian green mussel (*Perna viridis*) is known to be a fast-growing bivalve widely distributed in the tropical, subtropical coastal and estuarine areas of the Asian–Pacific regions. Asian green mussels belonging to the Mytilidae family are known to be filter feeders and can filter organic particles, plant plankton, animal plankton, and microorganisms. With a protein content of 36.15%, mussel is considered to be a cheap source of protein for coastal livelihood [10]. The *Vibrio* species is the leading marine bacterium that causes infection in economically significant baby clams. The crucial element for pathogenesis is host resistance [11]. Thongchan et al. [12] observed a high population of *Vibrio* strains in blood clams and mussels. Due to disease outbreaks and environmental contamination, clam aquaculture is encountering severe issues [13]. Antibiotic therapy is a predominant treatment for bacterial infection, but the rising global concern is antibiotic-resistant bacteria. Multidrug-resistant isolates exhibit hemolysis-associated genes, specific virulence traits, and biofilm potential. The biofilm potential of an isolate enables it to adhere to surfaces and spread out, resulting in the formation of multicellular consortia. The three-dimensional structure of the biofilm can shield the cells under extreme environmental stress [14]. As mentioned by the Centers for Disease Control and Prevention, antibiotics, antibodies, and disinfectants are less effective against bacterial biofilms [15]. Thus, the necessary action should be taken to control various pathogenic bacteria via thermal or non-thermal processing and the application of natural antimicrobial agents.

Among those natural antimicrobial agents, marine chitooligosaccharides (COSs) have been known for their broad antibacterial, antifungal, and antiviral activities. In general, COSs with a low molecular weight (<10 kDa) can bind to negatively charged bacterial cell walls, causing cell damage and nutrient leakage. They can also penetrate bacterial cells and inhibit DNA replication [16]. Furthermore, their activities have been enhanced via conjugation with phenolic compounds, among which the COS–epigallocatechin gallate (EGCG) conjugate showed excellent antimicrobial activity, in which, synergistically, EGCG could interact with bacterial cell walls and proteins, causing nutrient leakage and inhibiting peptide transport [17]. For example, Buatong et al. [18] reported that COS-EGCG conjugate effectively inhibited spoilage bacteria and foodborne pathogens such as *Pseudomonas aeruginosa* and *Escherichia coli*, respectively.

Thus, the present study aimed to isolate, characterize, and identify *Vibrio* species from blood clams, baby clams, and Asian green mussels in the fresh market of Hat Yai, Songkhla Province, Thailand. A hemolysis test was conducted to detect the pathogenicity of *Vibrio* spp. associated with antibiotic susceptibility and biofilm formation. Moreover, the antimicrobial activity of COS-EGCG conjugate against *Vibrio* strains was also determined.

## 2. Materials and Methods

### 2.1. Chemical and Microbial Media

Microbial media, including thiosulfate citrate bile salt sucrose (TCBS) agar, alkaline peptone water (APW), Wagatsuma agar base medium, tryptone soy broth (TSB), and tryptone soy agar (TSA), were procured from Oxoid (Thermo Fischer Scientific, Waltham, MA, USA), except HiCrome^TM^ Vibrio agar, which was bought from HIMEDIA (Himedia laboratories, Maharashtra, India). Ethanol was acquired from RBI Labscan™ (Bangkok, Thailand).

### 2.2. Sample Collection and Vibrio Isolation

**Figure 1:**
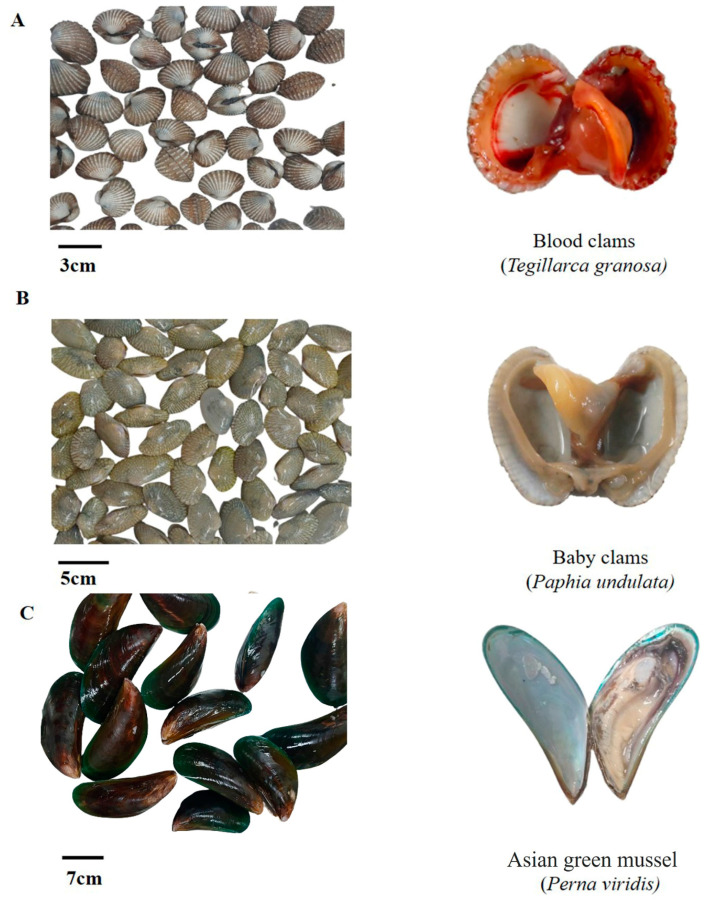
Blood clams (*Tegillarca granosa*) (**A**), baby clams (*Paphia undulata*) (**B**), and Asian green mussels (*Perna viridis*) (**C**) collected from local fresh markets in the Hat Yai district, Songkhla province, Thailand.

Blood clams (*Tegillarca granosa*) (Figure 1A), baby clams *(Paphia undulata*) (Figure 1B), and Asian green mussel (*Perna viridis*) (Figure 1C) were randomly selected. The samples were taken two times in only one month, and the second sampling was performed 15 days after the first sampling from the fresh market of the Hat Yai district, Songkhla Province, Thailand. In less than an hour, all the samples, in plastic bags under iced conditions, were carried to the seafood microbiology laboratory, PSU, Hat Yai, Thailand. The samples were rinsed in sterilized distilled water. The meat from the blood clams (BLs), baby clams (BBs), and Asian green mussels (Ms) was removed using an aseptic technique. Twenty-five grams of each sample was homogenized with 225 mL of alkaline peptone water (APW) (polypeptone, 10 g/L; NaCl, 20 g/L; pH 8.6) with the aid of a stomacher (Stomacher 400 Seaward Medicals, Worthing, UK) at 230 rpm for 1 min. APW is considered to be a good selective media. Two mixtures were prepared, out of which, one was utilized as a pre-incubated sample and the other was incubated for 6–8 h at 35 ± 2 °C for enrichment [14]. The culture from the pre-incubated sample was diluted from 10^−1^ to 10^−4^, and the APW-enriched culture was diluted from 10^−1^ to 10^−7^. Afterward, 100 µL of the diluted samples was dispersed on a thiosulfate citrate bile salt sucrose (TCBS) agar plate and incubated at 37 °C for 24 h. The green and yellow-colored colonies were selected and stored in 20% glycerol (*v*/*v*) in TSB with 3% NaCl (*w*/*v*) medium (TSB-3N). All *Vibrio* spp. were kept at −80 °C till further study.

### 2.3. Classification and Identification of Vibrio

The green and yellow colonies of *Vibrio* on TCBS agar were further cultured on an HiCrome^TM^ Vibrio agar plate, which was incubated under the same condition as TCBS agar plates. Based on the colony characteristics, all the isolates with similar characteristics on each media were grouped.

### 2.4. Pathogenicity Detection of Vibrio Isolates

A hemolytic test on a blood agar medium was used for the pathogenicity detection of *Vibrio* isolates [19]. A hemolysis assay was carried out using Wagatsuma agar base medium containing 5% human erythrocyte. Erythrocytes were washed with cold phosphate-buffered saline (PBS) solution three times and centrifuged at 4000× *g* at 4 °C for 5 min. The erythrocyte pellet was resuspended in PBS before use. *Vibrio* cultures in TSB-3N medium were adjusted to a 0.5 McFarland standard (~10^8^ CFU/mL) with NSS after 24 h of incubation at 37 °C. Thereafter, 2 µL of the culture was inoculated on Wagatsuma blood agar (WBA) plates and then subjected to incubation at 37 °C for 24 h [20]. The *β*-hemolysis-positive *Vibrio* strains were selected for identification using Matrix-Assisted Laser Desorption/Ionization Time-of-Flight Mass Spectrometry (MALDI-TOF MS) (MALDI-Biotyper^®^ system, microflex LT; Bruker Daltonik GmbH, Bremen, Germany) as described previously by Moussa et al. [21].

### 2.5. Biofilm Formation

For the biofilm formation test, the crystal violet (CV) staining method was adopted [22]. Overnight cultures of *β*-hemolysis-positive *Vibrio* strains were diluted 50-fold using 200 µL of TSB-3N medium in a 96-well plate and incubated at 37 °C for 48 h. The planktonic cells were transferred to the new 96-well plate to measure the bacterial cell density at 600 nm (A_600_). Thereafter, the culture plate was washed with 200 µL of sterile PBS solution 3 times, and the adhered bacterial cells were stained with 200 µL of 0.1% CV solution for 15 min. The wells were thoroughly washed with 200 µL of sterile distilled water thrice and then dried under laminar flow for 4 h. To solubilize the bound dye in each well, 200 µL of ethanol was added to the wells. The absorbance at 570 nm (A_570_) was read. The relative biofilm formation index (*BFI*) was determined by the following equation:

$$BFI=\frac{AB-CW}{GB-GW}$$

where *AB* is the A_570_ of CV-stained biofilm forming on the wells after 48 h, *CW* is the A_570_ of CV-stained blank wells considered as a control containing only TSB-3N, *GB* is the A_600_ of the bacterial culture in the wells after 48 h, and GW is the A_600_ of TSB-3N without bacteria. The degree of biofilm production was characterized as non-biofilm forming (*BFI* < 0.35), weak biofilm forming (0.35 ≤ *BFI* ≤ 0.69), moderate biofilm forming (0.70 ≤ *BFI* ≤ 1.09), and strong biofilm forming (*BFI* ≥ 1.10) [23].

The BFI determines the ability for biofilm formation and their production dynamics over time, the biofilm quantity, and the level of biofilm dispersal [24].

### 2.6. Detection of Virulence Genes

Three virulence genes, including thermostable direct hemolysin (*tdh*), *tdh*-related hemolysin (*trh*), and thermolabile hemolysin (*tlh*), were selected for detection among all the *β*-hemolysis-positive *Vibrio* isolates using specific primers [25]. A *V. parahaemolyticus* HVP2 strain gifted by the Faculty of Medicine, PSU, Hat Yai, was used as a positive control, and sterile distilled water was used as a negative control for the aforesaid virulence genes. Bacterial DNA extraction was performed using a DNeasy UltraClean Bacteria kit (Qiagen DNeasy^®^ UltraClean^®^ Microbial Kit, Germany) according to the manufacturer’s protocol. The genomic DNA quality was measured using a NanoDrop Lite Plus Spectrophotometer (Thermo Fisher Scientific, Wilmington, DE, USA). PCR reaction mixture and condition tests were performed to detect the *tdh*, *trh*, and *tlh* genes described by Palamae et al. [14]. The specific primers of the virulence genes are shown in Table 1. PCR products were measured on 2% agarose gel electrophoresis and stained with 1 µg/mL ethidium bromide to measure the band under UV light.

### 2.7. Antibiotic Susceptibility

The antibiotic susceptibility of all *β*-hemolysis-positive *Vibrio* isolates was determined using the Sensititre^TM^ broth microdilution system (Trek Diagnostic Systems, Cleveland, OH, USA) [26]. *Vibrio* isolates were cultured on TSA-3N agar and incubated at 37 °C overnight. After incubation, the bacterial cultures were suspended with sterile NSS to obtain turbidity equal to the 0.5 McFarland standard. Each bacterial suspension (100 µL) was transferred to cation-adjusted Mueller Hinton broth (MHB) for 10 mL. Then 100 µL of the mixture was transferred into each well of the CML1FMAR custom MIC plates (Trek Diagnostic Systems Inc., Cleveland, OH, USA) containing 21 different antibiotics, including amikacin (AN), amoxicillin/clavulanic acid (AMC), ampicillin (AM), ampicillin/sulbactam (SAM), cefepime (FEP), cefotaxime (CTX), cefoxitin (FOX), ceftazidime (CAZ), ceftriaxone (CRO), cefuroxime (CXM), ciprofloxacin (CIP), colistin (CL), doripenem (DOR), ertapenem (ETP), gentamicin (GM), imipenem (IPM), levofloxacin (LVX), meropenem (MEM), netilmicin (NET), piperacillin/tazobactam (TZP), and trimethoprim/sulfamethoxazole (SXT) to obtain the final concentration of 200 µL. The panels were sealed and incubated at 35 ± 2 °C for 18 h [27]. The MIC values were measured after incubation. The MIC value of the antibiotic was the lowest concentration of antibiotic that inhibits the growth of bacteria. There are four categories for the interpretation of the antibiotic susceptibility test: susceptible (S), intermediate (I), resistant (R), or, in some cases, no interpretation (NI). NI indicates that the MIC could not be determined at the concentrations of the antibiotic used. The results of the MIC were interpreted using approved clinical resistance breakpoints following the Clinical and Laboratory Standards Institute (CLSI) document M45 [27]. The bacteria that showed resistance to two or more antibiotics were defined as multidrug resistance (MDR) strains.

### 2.8. Bacterial Identification

The *β*-hemolysis-positive and multidrug-resistant *Vibrio* isolates were selected for identification using *16s rRNA* gene sequencing. The PCR reaction was performed in an Eppendorf™ Mastercycler™ Nexus Thermal Cycler (Eppendorf, Hamburg, Germany) using a universal primer specific to the *16s rRNA* gene (Table 1). The genomic DNA was diluted to 50 ng/µL to be subject to PCR reaction. The conditions for the amplification of the *16s rRNA* gene were considered, following Pascual et al. [28]. The PCR products were further purified using a PureLink^TM^ Quick PCR Purification Kit (Invitrogen, Thermo Fischer Scientific, Baltics, NA, USA). The purified PCR products were sequenced directly by Macrogen Inc., Korea, using universal primers. The consensus sequences were compared to the sequences of type strains of the bacteria in the EzBioCloud database using a 16s-based ID feature for searching the most similar species based on *16s rDNA* sequences. The multiple alignment of sequences was analyzed using the ClustalW of the MEGA version 11 software package [29]. A phylogenetic tree was constructed using the distance neighbor-joining (NJ) method with 1000 bootstrap replicants.

### 2.9. Effect of COS-EGCG Conjugate on Inhibition of MDR Vibrio Strains

#### 2.9.1. Preparation of COS-EGCG Conjugate

COS–EGCG was prepared using the AsA/H_2_O_2_ redox pair method as mentioned by Mittal et al. [16].

#### 2.9.2. Minimum Inhibitory Concentration (MIC) and Minimum Bactericidal Concentration (MBC)

The MIC and MBC of the COS-EGCG conjugate against five MDR *Vibrio* strains (BL99, BL105, BL186, M34, and M105) were determined in a 96-well plate using a colorimetric broth microdilution [18]. The COS-EGCG conjugate powder was dissolved with 0.01% acetic acid and sterilized by filtration using a 0.22 µm Millipore filter. The COS-EGCG conjugate solution was diluted with Mueller Hilton broth (MHB) (Difco^TM^, Baltimore, MD, USA) containing 3% NaCl (*w*/*v*), using a two-fold serial dilution method in a 96-well plate in triplicate to obtain ten various concentrations (50 µL/well). All five isolates were cultured in TSB with 3% NaCl and incubated at 37 °C for 18 h. The inoculum was prepared with 0.85% NSS to obtain a bacterial turbidity to equal the 0.5 McFarland standard (~1.5 × 10^8^ CFU/mL) and then diluted with TSB-3N to obtain 1.5 × 10^6^ CFU/mL. The inoculum was added to each well with 50 µL. The final concentrations of COS-EGCG conjugate were in a range of 4–2048 µg/mL. The 96-well plate was incubated for 15 h, and then 20 µL of 0.18% resazurin dye solution was added to each well. The 96-well plate was incubated for 3 h according to the same conditions. The lowest concentration of COS-EGCG conjugate with a blue/purple color in the wells was recorded as the MIC value. All the blue/purple-color wells were selected to determine the MBC value on Mueller Hilton agar (MHA) (Difco^TM^, Baltimore, MD, USA) added with 3% NaCl (*w*/*v*) by drop plate method with 2 µL. After incubation at 37 °C for 18 h, the lowest concentration of COS-EGCG conjugate in which *Vibrio* colony was not detected on the MHA with a 3% NaCl plate was recorded as the MBC value. Positive and negative controls were prepared using 0.01% acetic acid in MHA containing 3% NaCl with and without bacteria, respectively.

#### 2.9.3. Scanning Electron Microscopy (SEM)

The effect of the COS-EGCG conjugate on the morphology of the five MDR *Vibrio* spp. was visualized using an FEI quanta 400 scanning electron microscope (FEI, Brno, Czech Republic). The inoculum of these isolates was prepared with 0.85% NSS to obtain 10^6^ CFU/mL after incubation in TSB with 3% NaCl at 37 °C for 18 h. Thereafter, the inoculum was treated with COS-EGCG conjugate at the final concentration of 4× MIC, followed by incubation at 37 °C for 18 h. The inoculum without any treatment was considered as a control. The treated culture was further centrifuged at 3000× *g* for 5 min, and the obtained cell pellet was then resuspended with 0.1 M phosphate buffer (pH 7.2). Furthermore, fixation and dehydration were carried out as mentioned by Buatong et al. [18].

### 2.10. Statistical Analysis

A completely randomized design (CRD) was used throughout the investigation. The analysis was replicated three times (*n* = 3).

## 3. Results and Discussion

### 3.1. Morphological Identification of Vibrio spp. Colonies

All samples, including blood clams (*n* = 2), baby clams (*n* = 2), and Asian green mussels (*n* = 2), were used for *Vibrio* isolation. Six hundred and forty-nine *Vibrio* isolates were obtained from the aforesaid samples on TCBS agar (Table 2). The colony colors on the TCBS agar included green colonies (237 isolates) and yellow colonies (412 isolates). Based on the colony color from the HiCrome^TM^ Vibrio agar, the *Vibrio* isolates were classified into five groups, including creamy white, fluorescent green, dark green, green, and purple (Table 2). The group representative isolates of these six groups were randomly selected to be identified by MALDI Biotyper^®^ analysis. The identification of *Vibrio* isolated from mollusks using the MALDI-TOF-MS tool has been reported [21,30]. Isolates from blood clams, baby clams, and Asian green mussels having green-colored colonies on TCBS and with creamy white, dark green, and purple-colored colonies on HiCrome^TM^ Vibrio agar were identified as *V. harveyi*, *V. parahaemolyiticus*, and *V. vulnificus,* respectively, while the yellow-colored colonies on TCBS with creamy white, fluorescent green, and light green-colored colonies on HiCrome^TM^ Vibrio agar were identified as *V. navarrensis, V. fluvialis,* and *V. alginolyticus,* respectively (Table 2). Both colored colonies were round in shape, with a diameter of 2–3 mm. The dominant species of *Vibrio* in blood clams and baby clams were *V. alginolyticus*, *V. navarrensis*, and *V. parahaemolyiticus*. The dominant species, including *V. diabolicus, V. alginolyticus, V. parahaemolyticus,* and *V. harveyi,* have been reported [31]. Furthermore, *V. parahaemolyiticus*, *V. fluvialis*, and *V. navarrensis* were the dominant species in Asian green mussels (Table 2). Compared to other previous reports of *Vibrio* species in Asian green mussels (*Perna viridis*), *V. alginolyticus* was the most abundant species, followed by *V. parahaemolyticus* and *V. cholerae*, respectively [32]. However, the dominant species of *Vibrio* in baby clams has never been reported; this study could be the first report of *Vibrio* spp. in baby clams.

### 3.2. Hemolysis

**Figure 2:**
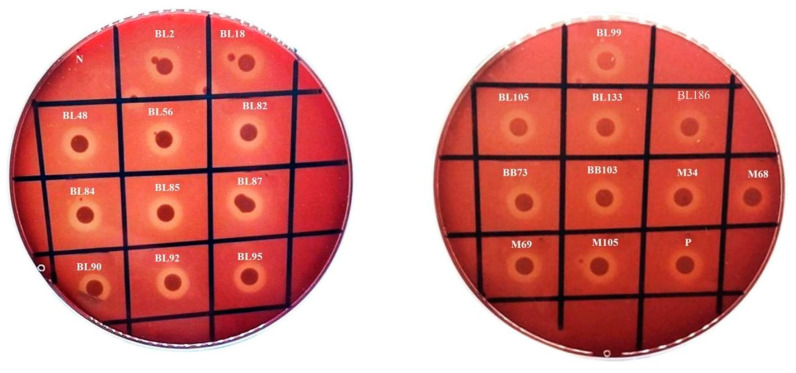
The *β*-hemolysis-positive 21 *Vibrio* strains on 5% sheep erythrocyte blood agar plate after incubation at 37 °C for 24 h. The labels on the plates represent the strains of *Vibrio* spp., NSS was used as negative control (N), and the clinical strain of *V. parahaemolyticus* strain HVP2 was used as positive control (P). BL, BB, and M: *Vibrio* strains from blood clams, baby clams, and Asian green mussels, respectively.

Normally, hemolysin is a type of exotoxin capable of rupturing cell membranes, resulting in the erythrocyte membrane lysis with the liberation of hemoglobin known as hemolysis [33]. There are three different varieties of hemolysis, including α-hemolysis (some blood cells are seen in the hemolysis zone), *β*-hemolysis (a clear zone is observed around the expanding colonies, resulting in hemolysis), and γ-hemolysis (no clear zone or blood hemolysis is visible) [8]. The majority of the isolates (*n* = 371; 57.20%) showed γ-hemolysis, indicating the absence of hemolysis, followed by α-hemolysis (*n* = 257; 39.60%), in which hemoglobin in red blood cells was oxidized to methemoglobin. Moreover, 21 isolates (3.2%) showed *β*-hemolysis (Figure 2), indicating true hemolysis [34]. The highest number of *β*-hemolysis-positive isolates was found in blood clams, followed by Asian green mussels and baby clams. Based on the colony characteristics obtained on TCBS agar and HiCrome^TM^ Vibrio agar plates, all 21 isolates were identified, followed by confirmation by MALDI Biotyper^®^ analysis (Table 3). The result suggested the abundance of *V. alginolyticus* in blood clams and baby clams, whereas Asian green mussels showed an abundance of *V. parahaemolyticus*, considered to be a major cause of pathogen infection in seafood products. It is usually associated with cases of diarrhea and is opportunistically infective to humans, causing wound infections in patients through soft tissue, ear, and skin lesions debased by seawater [35]. According to Hikmawati et al. [32], *V. parahaemolyticus* showed the Kanagawa phenomenon (*β*–hemolysis) in 57% of cases, indicating that the isolates were capable of hemolyzing human erythrocytes and providing a risk to those who ingest raw or uncooked mollusks. In comparison to bacteria without *β*-hemolysis, those with *β*–hemolysis might multiply more quickly in the gastrointestinal system. This ability plays a crucial role in determining the virulence of a particular *Vibrio* species. The pathogenicity can be determined by the generation of enterotoxin during both *β*–hemolysis and α-hemolysis. The strain that has *β*–hemolysis can persist longer than that without *β*–hemolysis [32].

### 3.3. Presence of Virulence Gene

**Figure 3:**
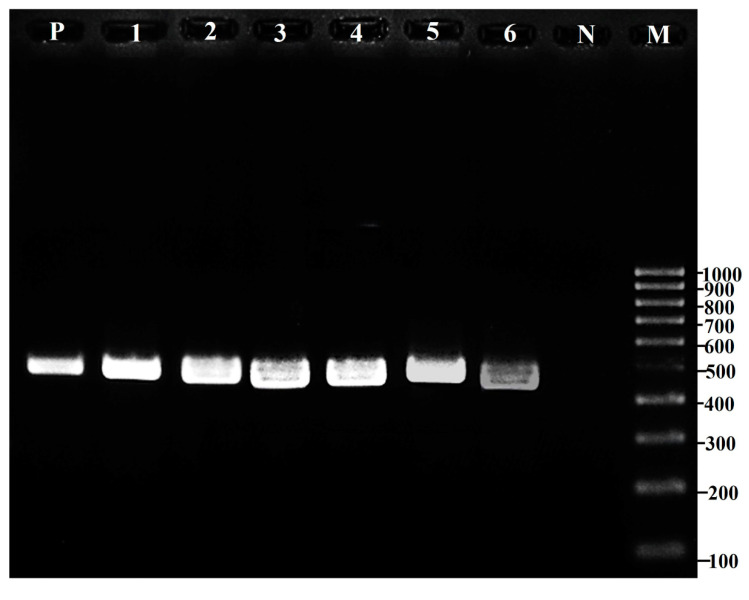
Amplification of *tlh* gene in *β*-hemolysis-positive *Vibrio* spp. using specific primers. Lane P: positive control, Lanes 1–6: represent *Vibrio* spp. strains BL18, BL82, BL84, BL85, BL90, and BL92, respectively, Lane N: negative control, Lane M: 100 bp Ladder. For the captions, see Figure 2.

For further confirmation of the 21 *β*-hemolysis-positive isolates, the virulence genes encoding the *tdh, tlh*, and *trh* genes were observed by DNA extraction of the isolates, followed by PCR amplification. Gel electrophoresis for the final PCR products was performed, to confirm the presence of the genes encoding *tdh*, *tlh*, and *trh* (Figure 3). The *trh* gene is a heat-labile toxin, which is immunologically related to *tdh,* and both of these genes have a similar homology (70%). The *tdh* gene is a pore-forming toxin that can produce pores in erythrocyte membranes. Water and ions could easily pass due to their relatively high pores [36]. The *tlh* genes can encode the *Vibrio* enterotoxin called thermoliable hemolysin (*tlh*), which can lyse the blood cells. All the clinical and environmental strains of *V. parahaemolyticus* carry the *tlh* gene, so it is markedly more common in situations and responsible for intestine infection [37]. Based on this observation, six isolates (BL18, BL82, BL84, BL85, BL90, and BL92) isolated from blood clams among the 21 *β*-hemolysis-positive isolates were confirmed to be *V. parahaemolyticus,* as indicated by a positive response (*tlh+*), whereas all the other *Vibrio* spp. isolates from baby clams and Asian green mussels indicated a negative response (*tlh-*), indicating them as non-*V. parahaemolyticus* isolates (Figure 3). Malcolm et al. [38] reported thatthe blood clams purchased from retailers in Malaysia were stated to have a high incidence of pathogenic *V. parahaemolyticus*, which may be an important microbiological hazard linked to the consumption of raw or undercooked products. *V. parahaemolyticus* has been identified by PCR using the *tlh, tdh,* and *trh* genes in the Middle Black Sea Coast of Turkey. Similarly, a species-specific gene called *tlh* was used to detect complete *V. parahaemolyticus*, and the hemolysin genes *tdh* and *trh* were used to identify the virulent strains [39]. It was also discovered that the majority of *V. parahaemolyticus* strains recovered from seafood samples lack the *tdh* and *trh* genes. However, *V. parahaemolyticus* has a complicated and interacting pathogenicity [39].

### 3.4. Biofilm Formation

**Figure 4:**
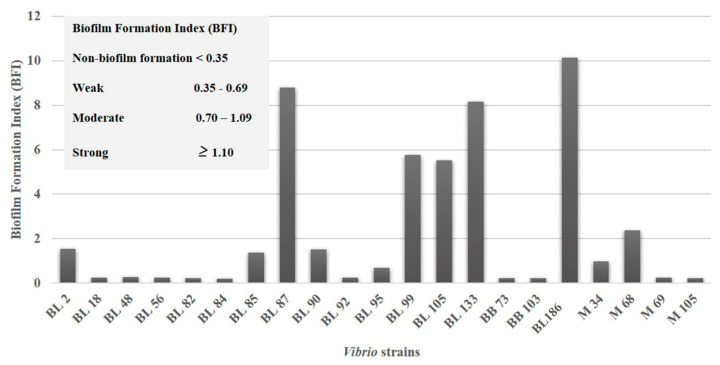
Biofilm formation index (BIF) of the *β*-hemolysis-positive 21 *Vibrio* strains. For the captions, see Figure 2.

Out of 21 *β*-hemolysis-positive *Vibrio* strains, only nine isolates, out of which eight were isolated from blood clams (BL2, BL85, BL87, BL90, BL99, BL105, BL133, and BL186) and one from Asian green mussels (M68), showed a strong biofilm formation as advocated by the BFI (Figure 4 and Table 2), whereas one isolate from blood clam (BL95) and one from Asian green mussels (M34) showed a moderate biofilm formation. The remaining six isolates from blood clams (BL18, BL48, BL56, BL82, BL84, and BL92), two isolates from baby clams (BB73 and BB103), and two isolates from Asian green mussels (M69 and M105) showed non-biofilm formation based on the BFI. Amongst all the other isolates, BL186, corresponding to *V. fluvialis,* showed a higher biofilm formation ability than the remaining eight isolates. Biofilm is a major contributor to persistent and recurring infections caused by clinically significant pathogens globally and resistant to antibiotic therapy. This is because the development of biofilms and the subsequent encapsulation of bacterial cells in a complicated matrix might increase bacterial resistance to antimicrobials and sterilizing treatments, making it challenging to eliminate and manage these organisms [40]. To adhere to the surfaces of foods, microorganisms can form biofilms by secreting extracellular polymers to encapsulate themselves in an extracellular matrix. Reversible attachment, irreversible adhesion, early biofilm structure development (the formation of tiny colonies), biofilm maturation, cell separation, and diffusion are the five sequential processes in the formation of a biofilm [41]. By employing an altered metabolism, gene expression, and protein production within the biofilm, the bacteria can adjust to environmental anoxia and nutritional limitations, which can result in a slower metabolic rate and a slower rate of cell division. Overall, these modifications make the bacteria more resistant to antimicrobial therapy [42].

### 3.5. Antibiotic Resistance of Selected Isolates

*β*-hemolysis-positive isolates were analyzed against 21 different antibiotics. There were noticeable differences in susceptibility toward the 21 antibiotics for these isolates (Table 4). Out of the 21 isolates, 4 isolates (BL99, BL105, M34, and M105) were resistant to CTX and CXM. Moreover, the BL186 isolate was resistant to six antibiotics, including AMC, SAM, CTX, CXM, MEM, and SXT. Antibiotic resistance in *Vibrio* is getting worse and is currently one of the main risks to public health and global aquaculture. A danger to human health could be indicated by the presence of possibly resistant and pathogen-related taxonomic groupings (*Acinetobacter*, *Arcobacter*, and *Clostridium*) (Huang et al., 2022). Gxalo et al. [43] observed that *Vibrio* species exhibited an AMC resistance greater than 85%. Cefuroxime (CXM), a third-generation antibiotic, likewise showed a resistance of more than 75% among the *Vibrio* isolates, and the results were comparable as reported by Gxalo et al. [43]. Biofilm-forming bacteria are extremely resilient to changing conditions, including detergents and antibiotics [44]. A high resistance to drugs can be the result of a high ability to form biofilms. Among strong biofilm-forming isolates, BL99, BL105, and M34 were also resistant to antibiotics. Other isolates were unable to form biofilms and were consequently susceptible to antibiotics. A bacterium can create a biofilm to protect itself from harmful environmental factors, including antibiotics and antimicrobial compounds [45]. Based on the MDR analysis, Asian green mussels and blood clams possessed MDR strains, whereas no such strain was found in the baby clams. In Thailand, people prefer consuming bivalve mollusks, especially blood clams and Asian green mussels. The most MDR strains were found in *Vibrio* spp. obtained from blood clams, according to our study. Consequently, an emphasis will be placed on investigating the antibiotic susceptibility of *Vibrio* spp. isolated from blood clams and Asian green mussels. Obtaining more MDR strains is probable when there are more samples. Furthermore, further research on the antibiotic resistance genes in resistant strains will be studied. The selected MDR strains, namely, BL99, BL105, BL186, M34, and M105, were further identified and treated with the COS-EGCG conjugate.

### 3.6. Molecular Identification Using 16s rRNA Gene Sequencing

**Figure 5:**
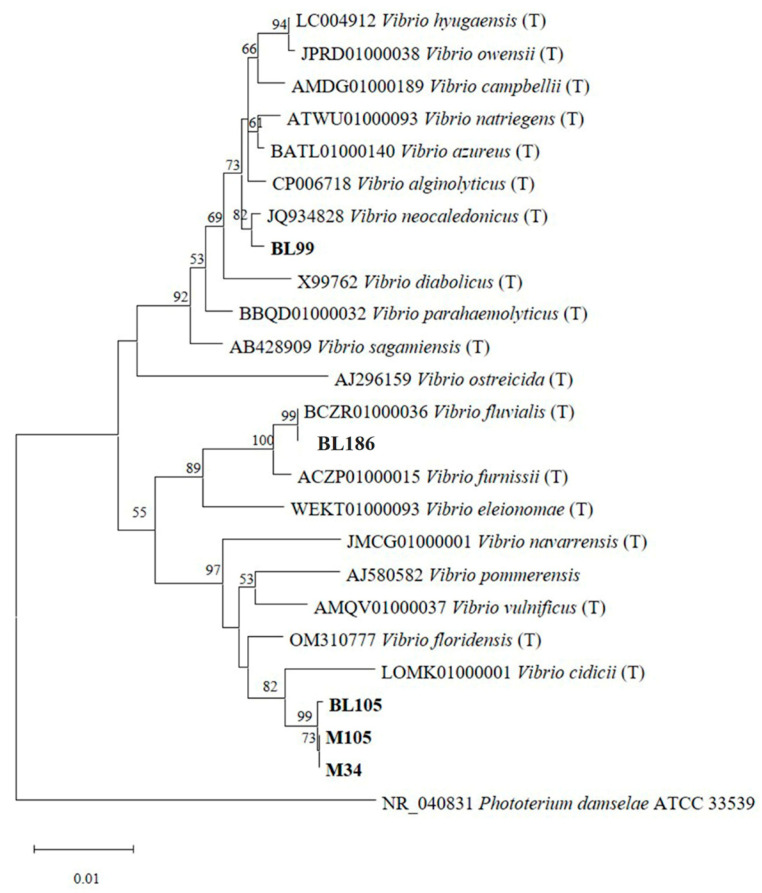
Phylogenetic tree of five MDR *Vibrio* spp. isolated from blood clams (BLs) and Asian green mussels (Ms) based on partial *16S rRNA* gene sequences. The tree was constructed by the neighbor-joining method using MEGA 11 software. The number on the node of the tree represents the bootstrap value (%) from 1000 replications. *Photobacterium damselae* ATCC 33539 was used as an outgroup. For the captions, see Figure 2.

The five strains of MDR *Vibrio* were subjected to identification based on partial sequences of the *16s rRNA* gene. The nucleotide sequences of the MDR *Vibrio* strains were compared with the related species of strains from EZBioCloud databases. The phylogenetic tree was constructed by MEGA 11 using the neighbor-joining method, which showed that strain BL186 was grouped in the same clade with *V. fluvialis* and showed a 100% sequence similarity with *V. fluvialis* BCZR01000036 (Figure 5). Therefore, the strain BL186 was identified to be *V. fluvialis,* which is a newly recognized foodborne pathogen becoming more prevalent worldwide. Moreover, many countries have reported diarrheal symptoms in travelers caused by *V. fluvialis*, mainly consuming fresh seafood [46]. *V. fluvialis* can produce various toxins and is important in pathogenesis, including enterotoxin-like substances, cytotoxin, and hemolysin [47]. The major hemolysin in *V. fluvialis* is VHF (*Vibrio fluvialis* hemolysin). This toxin causes a pore-formation and induces osmotic lysis in the erythrocytes of humans [47]. *V. fluvialis* strain BL186 was isolated from blood clams and showed resistance to six antibiotics. The resistance genes can be transferred from Gram-negative bacteria to *Vibrio* spp. by plasmid, transposons, integrons, and the SXT element [47]. The SXT element transferred from *V. cholerae* to *V. vunificus* has been reported. Therefore, the *V. fluvialis* strain BL186 may receive the resistance gene from other *Vibrio* spp. of the same habitat. The BL99 strain was closely related to *V. neocaledonicus* JQ934828, with a high bootstrap value (Figure 5). When considering the nucleotide sequence, the strain BL99 showed the most similarity to *V. neocaledonicus* JQ934828, with a 99.78% similarity. Therefore, the strain BL99 was identified to be *V. neocaledonicus*. Strains M34, M105, and BL105 belonged to *V. cidicii* LOMK01000001, with the sequence similarity between 98.65 and 98.78%. Therefore, strains M34, M105, and BL105 were identified as *V. cidicii*. Although strain BL99 was identified as *V. alginolyticus*, strains M34, M105, and BL105 were identified as *V. navarensis* using the MALDI-Biotyper^®^. The sequence similarity results were different. However, the misidentification of *Vibrio* spp. from marine mollusks using the MALDI-Biotyper^®^ database has been reported. *V. jasicida* has been misidentified as *V. harveyi* with Bruker’s database, even though the matching score was higher than 2 [21]. Furthermore, the identification of *Vibrio* by MALDI-TOF-MS is dependent on the database in the machine (Moussa et al., 2021). Even though MALDI-TOF-MS analysis is the rapid method for *Vibrio* identification, it can cause some misidentifications for some species. Therefore, molecular identification based on the *16s rRNA* gene sequence or some housekeeping genes (*hsp60*, *gyrB*, *topA*, *pyrH*, *ldh*, *mreB*, *gapA*, *ftz*, *dnaJ*) is necessary to confirm the species level of some *Vibrio* [21].

### 3.7. Antimicrobial Activity of COS-EGCG Conjugate against Multidrug-Resistant (MDR) Vibrio Strains

**Figure 6:**
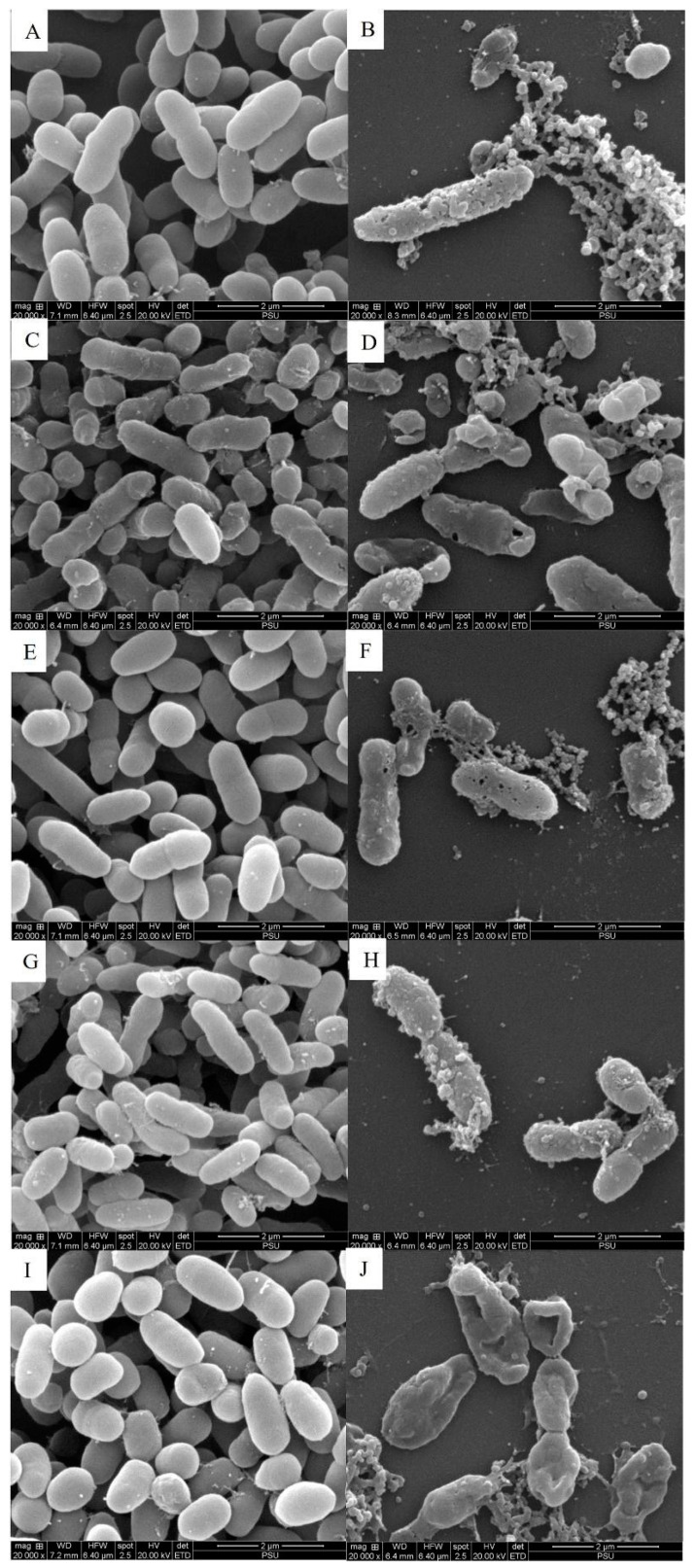
Morphological changes in MDR *Vibrio* spp. isolates treated with COS-EGCG conjugate at the final concentration of 4 × MIC (**B**,**D**,**F**,**H**,**J**) and untreated cells as the control (**A**,**C**,**E**,**G**,**I**). SEM images of *Vibrio neocaledonicus* strain BL99 (**A**,**B**); *Vibrio cidicii* strain BL105 (**C**,**D**); *Vibrio cidicii* strain M34 (**E**,**F**); *Vibrio cidicii* strain M105 (**G**,**H**); and *Vibrio fluvialis* strain BL186 (**I**,**J**).

The antimicrobial effect of COS-EGCG conjugate against the MDR *Vibrio* strains was determined. The lowest inhibitory activity was found in *Vibrio* spp. strains BL99 and M105, with a MIC and MBC of 128 µg/mL (Table 5). On the other hand, COS-EGCG conjugate showed the highest antimicrobial activity against *Vibrio* spp. strains BL186 and M105, with a MIC and MBC of 64 µg/mL. The BL105 strain showed a MIC and MBC of 64 and 128 µg/mL, respectively. Thus, COS-EGCG conjugate showed bactericidal effects (MBC/MIC ratio ≤ 4) on MDR *Vibrio* strains (Table 5). Singh et al. [17] reported that EGCG is known to exhibit antimicrobial activity against several Gram-negative and Gram-positive bacteria, causing membrane breakdown, bacterial DNA gyrase inhibition, and DNA supercoiling prevention, leading to bacterial cell death. The increasing antimicrobial action of COS was enhanced by EGCG conjugated with COS. The antimicrobial activity of COS-EGCG conjugate was supported by the damaged cell structure and morphological changes of the MDR *Vibrio* strains (Figure 6A–J). When compared to the untreated cells or controls having a smooth outer cell surface with an intact membrane, the treated cells showed cell disruption, with pores and degeneration of the bacterial cell wall. The pores in the treated cells might lead to the exudation of cell components, resulting in the penetration of COS-EGCG conjugate into the bacterial cell, thus inhibiting the growth of bacteria. It was also observed that the treated cells had traces of COS-EGCG conjugate attached to the cell surface, as well as the outer surface. Moreover, the cell density for each treated sample was less, as compared to that of the untreated cells or control, depicting the effectiveness of COS-EGCG conjugate on the cells. That EGCG causes cell death by destroying the bacterial cell and inhibiting various extracellular and intracellular enzymes produced by bacteria has been reported [48]. An intracellular enzyme known as AKP is located between the cell membrane and cell wall in the majority of bacteria. Pei et al. [49] reported that the activity of AKP was increased by the action of EGCG, which was due to cell wall integrity disturbance. This resulted in the release of AKP into the external environment. Moreover, COS penetrates to the cell and blocks DNA and RNA transcription when the negatively charged bacterial cell binds with the positively charged COS, resulting in the absorption of COS [50]. Thus, the conjugation of COS and EGCG causes the deformed and distorted structure of the cells. The pores generated favor the leakage of nutrients, leading to cell lysis.

## 4. Conclusions

*Vibrio* spp. isolated from blood clams, baby clams, and Asian green mussels were isolated and identified based on their morphology and molecular characterization. From the MALDI-TOF-MS profiles, most *Vibrio* species in blood clams, baby clams, and Asian green mussels were identified to be *V. alginolyticus* in abundance, followed by *V. parahaemolyticus* and *V. navarrensis.* Out of 649 isolates, 21 isolates were found to be pathogenic, as they were *β*-hemolysis-positive. The majority of these isolates were isolated from blood clams. Isolates from baby clams were less in number and showed no pathogenicity (MDR strains were not found) compared to blood clams and Asian green mussels. Six *tlh*-positive *Vibrio* isolated from blood clams were found from the 21 isolates of *β*-hemolysis-positive but did not show a positive result for *tdh* or *trh* gene detection. The species confirmation of MDR *Vibrio* isolates was obtained after analyzing the *16s rRNA* gene sequence similarity of these isolates with the type strains. Three species of *Vibrio* were *β*-hemolysis-positive and resistant to antibiotics, including *V. fluvialis, V. neocaledonicus*, and *V. cidicii.* Furthermore, COS-EGCG conjugate showed strong bactericidal activity against those MDR strains. Although COS-EGCG conjugate showed the potential to lyse the MDR strains in in-vitro conditions, their activity in a real system should be determined, especially during the storage of mollusks. These findings highlighted the potential of various *Vibrio* spp. to contaminate seafood, especially clams, causing foodborne illness. Further treatment on inactivation is still required to ensure the safety of consumers, especially cytotoxicity testing. Moreover, the effects of COS-EGCG conjugate to reduce the contaminated *Vibrio* spp. in marine bivalve mollusk products will be studied.

## Figures and Tables

**Table 1:** Primers used for the detection of virulence genes and *16s rRNA* gene amplification.

Target Genes	Primers	Sequence (5’-3’)	Amplicon Size (bp)	References
*tdh*	tdh-F	GTAAAGGTCTCTGACTTTTGGAC	269	Siddique et al. (2021) [25]
	tdh-R	TGGAATAGAACCTTCATCTTCACC		
*tlh*	tlh-F	AAAGCGGATTATGCAGAAGCACTG	450	
	tlh-R	GCTACTTTCTAGCATTTTCTCTGC		
*trh*	trh-F	TTGGCTTCGATATTTTCAGTATCT	500	
	trh-R	CATAACAAACATATGCCCATTTCCG		
*16s rRNA*	27F	AGAGTTTGATCCTGGCTCAG	1400	Palamae et al. (2022) [14]
	1429R	GGTTACCTTGTTACGACTT		

**Table 2:** Color characteristics of *Vibrio* colonies on TCBS and HiCrome^TM^ Vibrio agar and identification of *Vibrio* using MALDI Biotyper^®^ analysis.

TCBS	HiCrome^TM^ Vibrio	MALDI Biotyper^®^ Analysis	BL	BB	M	Total of Each Character
Yellow 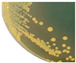	Creamy white 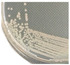	*V. navarrensis*	82	44	12	138
Yellow 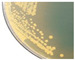	Indigo/Purple 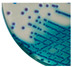	*V. fluvialis*	49	0	15	64
Yellow 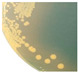	Light green 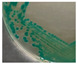	*V. alginolyticus*	123	80	7	210
Green 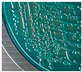	Light green 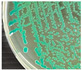	*V. parahaemolyticus*	73	41	47	161
Green 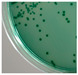	Purple 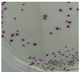	*V. vunificus*	14	22	0	36
Green 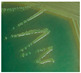	Creamy white 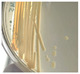	*V. harveyi*	16	21	3	40
Total						649

Where TCBS is thiosulfate citrate bile salt sucrose agar plates. BL, BB, and M are codes used for blood clams, baby clams, and Asian green mussels, respectively.

**Table 3:** Species identification of 21 positive *β*-hemolysis *Vibrio* isolates using MALDI Biotyper^®^ analysis.

*Vibrio* Strains	Species
BL2, BL48, BL56, BL87, BL95, BL99, BB73, BB103, M69, BL133	*Vibrio alginolyticus*
BL105, M34, M68, M105	*Vibrio navarrensis*
BL186	*Vibrio fluvialis*
BL18, BL82, BL84, BL85, BL90, BL92	*Vibrio parahaemolyticus*

For the captions, see Figure 2.

**Table 4:** Antibiotic resistance profiles of 21 *β*-hemolysis-positive *Vibrio* spp.

Antibiotics	Concentration (µg/mL)	*Vibrio* Strains							
		BL2, BL18, BL48, BL82, BL87, BL90, BL92, BL95, BL133, M68, M69	BL56, BL84, BL85, BB103	BB73	BL99	BL105	BL186	M34	M105
AN	8–32	S	S	S	S	S	S	S	S
AMC	4/2–16/8	S	S	S	S	I	**R**	I	I
AM	8–16	NI	NI	NI	NI	NI	NI	NI	NI
SAM	4/2–16/8	S	S	S	S	I	**R**	I	I
FEP	1–32	S	S	S	S	S	S	I	S
CTX	1–32	S	S	S	**R**	**R**	**R**	**R**	**R**
FOX	4–16	NI	NI	NI	NI	NI	NI	NI	NI
CAZ	1–32	S	S	S	S	S	S	S	S
CRO	0.5–32	NI	NI	NI	NI	NI	NI	NI	NI
CXM	8–16	S	I	NM	**R**	**R**	**R**	**R**	**R**
CIP	0.06–2	S	S	S	S	S	S	S	S
CL	1–8	NI	NI	NI	NI	NI	NI	NI	NI
DOR	0.5–16	NI	NI	NI	NI	NI	NI	NI	NI
ETP	0.5–4	NI	NI	NI	NI	NI	NI	NI	NI
GM	2–8	S	S	S	S	S	S	S	S
IPM	0.5–16	S	S	S	S	S	S	S	S
LVX	0.06–8	S	S	S	S	S	S	S	S
MEM	0.5–16	S	S	S	S	S	**R**	S	S
NET	8–16	NI	NI	NI	NI	NI	NI	NI	NI
TZP	8/4–64/4	S	S	S	S	S	S	S	S
SXT	1/19–4/76	S	S	S	S	S	**R**	S	S

S: susceptible; I: intermediate; R: resistant; NI: no interpretation; NM: not mentioned. For the captions, see Figure 2.

**Table 5:** Minimum inhibitory concentration (MIC) and minimum bactericidal concentration (MBC) of COS-EGCG conjugate against MDR *Vibrio* strains.

*Vibrio* Strains	MIC (µg/mL)	MBC (µg/mL)	MBC/MIC Ratio
*Vibrio neocaledonicus* BL99	128	128	1
*Vibrio cidicii* BL105	64	128	2
*Vibrio fluvialis* BL186	64	64	1
*Vibrio cidicii* M34	128	128	1
*Vibrio cidicii* M105	64	64	1

BL and M indicate isolates from blood clam and Asian green mussel, respectively.

## Data Availability

The original contributions presented in the study are included in the article; further inquiries can be directed to the corresponding author.
